# Enhanced Breast Cancer Diagnosis Using Multimodal Feature Fusion with Radiomics and Transfer Learning

**DOI:** 10.3390/diagnostics15172170

**Published:** 2025-08-28

**Authors:** Nazmul Ahasan Maruf, Abdullah Basuhail, Muhammad Umair Ramzan

**Affiliations:** Faculty of Computing and Information Technology, Department of Computer Science, King Abdulaziz University, Jeddah 21589, Saudi Arabia; abasuhail@kau.edu.sa (A.B.); muramzan@kau.edu.sa (M.U.R.)

**Keywords:** medical imaging, breast cancer, feature engineering, radiomics analysis, deep learning, transfer learning

## Abstract

**Background:** Breast cancer remains a critical public health problem worldwide and is a leading cause of cancer-related mortality. Optimizing clinical outcomes is contingent upon the early and precise detection of malignancies. Advances in medical imaging and artificial intelligence (AI), particularly in the fields of radiomics and deep learning (DL), have contributed to improvements in early detection methodologies. Nonetheless, persistent challenges, including limited data availability, model overfitting, and restricted generalization, continue to hinder performance. **Methods:** This study aims to overcome existing challenges by improving model accuracy and robustness through enhanced data augmentation and the integration of radiomics and deep learning features from the CBIS-DDSM dataset. To mitigate overfitting and improve model generalization, data augmentation techniques were applied. The PyRadiomics library was used to extract radiomics features, while transfer learning models were employed to derive deep learning features from the augmented training dataset. For radiomics feature selection, we compared multiple supervised feature selection methods, including RFE with random forest and logistic regression, ANOVA F-test, LASSO, and mutual information. Embedded methods with XGBoost, LightGBM, and CatBoost for GPUs were also explored. Finally, we integrated radiomics and deep features to build a unified multimodal feature space for improved classification performance. Based on this integrated set of radiomics and deep learning features, 13 pre-trained transfer learning models were trained and evaluated, including various versions of ResNet (50, 50V2, 101, 101V2, 152, 152V2), DenseNet (121, 169, 201), InceptionV3, MobileNet, and VGG (16, 19). **Results:** Among the evaluated models, ResNet152 achieved the highest classification accuracy of 97%, demonstrating the potential of this approach to enhance diagnostic precision. Other models, including VGG19, ResNet101V2, and ResNet101, achieved 96% accuracy, emphasizing the importance of the selected feature set in achieving robust detection. **Conclusions:** Future research could build on this work by incorporating Vision Transformer (ViT) architectures and leveraging multimodal data (e.g., clinical data, genomic information, and patient history). This could improve predictive performance and make the model more robust and adaptable to diverse data types. Ultimately, this approach has the potential to transform breast cancer detection, making it more accurate and interpretable.

## 1. Introduction

The most frequently diagnosed malignant neoplasm in women is breast cancer, which also stands as the second leading cause of cancer deaths in females worldwide [1,2]. Detecting the disease at an early stage is vital for better therapeutic outcomes and improved survival rates. An array of imaging techniques, including mammography, MRI, and ultrasonography, are fundamental to the detection of irregularities within breast tissue. Recent advancements have increasingly prioritized the application of medical imaging modalities for the early identification of breast neoplasms [3].

The advent of deep learning has significantly advanced medical imaging analysis, enabling precise and automated identification of breast malignancies. Convolutional neural networks (CNNs) and related deep learning frameworks excel in discerning high-dimensional feature representations in medical imaging, contributing to a reduction in diagnostic variability. In our previous study [4], a systematic review and meta-analysis investigated radiomics-driven deep learning and machine learning algorithms for breast cancer diagnosis, evidencing improved diagnostic precision and specificity, especially in mammographic and MRI-based imaging. Despite these advancements, significant limitations remain. Our previous study highlighted challenges such as heterogeneity in medical image features, limited availability of large annotated datasets, and limited model generalizability across heterogeneous imaging protocols and diverse patient cohorts, which pose a significant challenge.

Early identification of breast cancer faces significant challenges, primarily because of the shortage of manifold datasets necessary for effectively training predictive models. This limited representation can hinder the ability of models to generalize across different populations, resulting in inaccuracies. Additionally, class imbalances within the datasets can further exacerbate these issues, leading to biased predictions that may not accurately reflect the reality of all patient groups. As a result, these obstacles can compromise the effectiveness of early detection efforts for combating breast cancer.

Feature extraction is significant because it allows the analysis, measurement, and identification of distinct attributes within images that indicate the existence or advancement of cancer [5,6]. On the other hand, because of the complex and critical nature of medical data, medical image feature extraction requires innovative approaches [7]. Radiomics analysis is an effective feature engineering approach in medical image analysis that is crucial to improving patient outcomes. Recent studies [8,9,10,11] have conducted radiomic analysis and extracted radiomic features to advance medical image analysis. Radiomics techniques employ sophisticated algorithms to extract quantitative characteristics from medical images, such as the shape, texture, size, and intensity of tumors [12], which can then be used to develop prediction models for various medical image classification problems. Radiomics feature extraction, which provides interpretable, clinically relevant features, complements deep feature extraction, where deep learning models automatically derive hierarchical and abstract representations from imaging data. This synergy enhances diagnostic accuracy by combining the quantitative insights of radiomics into tumor characteristics with the ability of deep features to capture complex, nonlinear patterns [13].

The application of transfer learning, utilizing pre-trained deep neural networks such as VGG, ResNet, or Inception, empowers enhanced model performance by transferring complex feature extractors trained on vast, generic datasets to address the challenges of limited labeled medical imaging data. Integrating deep learning pretrained models with radiomics features addresses key challenges in breast cancer detection by leveraging their complementary strengths. Proper data augmentation and pre-trained models mitigate data limitations through transfer learning and extract complex, high-level features from images, whereas radiomics provides reproducible, standardized metrics to reduce subjectivity. This combination enhances the model accuracy, sensitivity, and specificity by integrating learned and handcrafted features into hybrid frameworks. Automating both approaches speeds up the analysis, minimizes time inefficiencies, and facilitates real-time application. This synergy enhances the reliability, consistency, and performance, advancing the precision and personalization of breast cancer detection.

Therefore, we propose a comprehensive methodology pipeline that integrates advanced data augmentation, rigorous multimodal feature engineering, and systematic evaluation of transfer learning models to optimize early breast cancer diagnosis. This research will lead to the following enhancements in performance and reliability:

- Advanced data augmentation techniques to tackle the issues of limited and imbalanced datasets, resulting in enhanced model robustness and generalization.
- Feature engineering by integrating multimodal features (radiomics and deep features) from the augmented training dataset. Radiomics feature selection was conducted using RFE (with random forest and logistic regression), ANOVA F-test, LASSO, and mutual information, alongside embedded approaches leveraging XGBoost, LightGBM, and CatBoost with GPU acceleration. The most informative features were subsequently integrated with deep features to build a unified multimodal feature space for final model development.
- An in-depth analysis of 13 transfer learning models, emphasizing their relative performance and illustrating significant enhancements in the accuracy of breast cancer diagnosis.

The remainder of this paper is organized as follows. Section 2 synthesizes the relevant literature and highlights critical gaps in current knowledge. Section 3 explains the research methodology utilized. Section 4 details the outcomes of the study concerning transfer learning models, comparing the findings with existing research. Section 5 discusses the findings while addressing the associated challenges and limitations. Finally, Section 6 summarizes the study and proposes future research pathways.

## 2. Literature Review

Khourdifi et al. [14] formed a deep learning methodology for the prompt identification of breast cancer, using radiomics and transfer learning techniques. Their method employed standardized preprocessing for mammographic images sourced from the CBIS-DDSM and INbreast datasets, enhancing both interpretability and robustness. The ensemble model achieved an impressive accuracy of 89.5%, along with a remarkable specificity of 90.2% when tested on the CBIS-DDSM dataset.

Pham et al. [15] introduced an architecture based on EfficientNet, incorporating image enhancement techniques to improve breast cancer prediction. They presented a fine-tuning procedure for pre-trained models, including ResNet-50, EfficientNet-B5, and Xception. The strategy incorporates a variety of preprocessing methods and leverages transfer learning to extract intrinsic markers, which positively contribute to improving model dependability and enhancing classification accuracy. The authors compared three ensemble methods—averaging, weighted averaging, and voting—achieving up to 91.36% accuracy for mass/calcification classification and 76.79% for benign/malignant classification.

Chugh et al. [16] introduced TransNet, a model that combines classical machine learning techniques with transfer learning paradigms. Their study demonstrated that transfer learning significantly enhances model generalizability, allowing for the detection of subtle features in mammograms. For feature extraction and classification, deep learning methods utilize neural network classifiers such as MobileNet, VGG16, VGG19, ResNet50, ResNet152, and DenseNet169. Among the evaluated models, MobileNet, ResNet50, and DenseNet169 achieved superior performance, each reaching an accuracy of 97% (±2%) on both the training and test sets.

Subha et al. [17] developed a depth-wise convolutional neural network (CNN) specifically designed for mammographic classification. Their study utilized pre-trained models, demonstrating enhanced efficiency in breast lesion classification. The proposed method achieved an accuracy of 95 percent.

Gujar et al. [18] employed radiomics-based convolutional neural network (CNN) models utilizing transfer learning for the classification of breast lesions. Their research demonstrated that the integration of radiomics features with deep learning significantly enhances both model sensitivity and specificity. Among the models tested, ResNet50 exhibited the highest performance, achieving a classification test accuracy of 0.72, and AlexNet attained a test accuracy of 0.64.

Alexandru et al. [19] compared the performance of fine-tuned deep learning models in classifying breast tissue from ultrasound images into multiple classes using transfer learning. In their study, the authors employed several transfer learning models combined with data augmentation. The highest performance of 98.11±0.10% was achieved using the DenseNet-121 model on their test set.

Asadulla et al. [20] proposed a novel approach for classifying breast cancer histopathology images. It improves interpretability and resilience by focusing on localized features and distinguishing problematic situations utilizing modified pre-trained CNN models and attention procedures. Transfer learning using five deep convolutional neural network models augmented by the convolutional block attention module is used. Testing accuracy rates for the attention mechanism (AM) utilizing the Xception model on the “BreakHis” breast cancer dataset are 0.992 and 0.995. The test accuracy for DenseNet121 using AMs is 0.996.

In a study by Francesca et al. [21], the authors developed and evaluated two deep learning models for the binary classification of breast lesions (benign vs. malignant) using digital breast tomosynthesis (DBT) images, with the goal of enhancing clinical decision-making and risk stratification. Both models utilized convolutional neural network (CNN) architectures, leveraging transfer learning with ImageNet pre-trained weights. The training and evaluation process employed a 10-fold cross-validation strategy combined with ensemble voting. The ResNet50-based model demonstrated an ROC–AUC of 63%, an accuracy of 60%, a sensitivity of 39%, and a specificity of 75% on the internal test set.

Wang et al. [22] proposed a cross-modality fusion approach combining mammography and ultrasound images using deep learning. Using three public datasets, they developed and compared three models: pre-trained CNNs with machine learning classifiers, transfer learning-based CNNs, and a custom 17-layer CNN. Evaluation based on accuracy and Kappa score showed that the custom CNN achieved the best performance with an accuracy of 96.4% and a Kappa of 0.927.

Xiong et al. [23] introduced HCMNet, a hybrid architecture that combines CNN and Mamba to enhance breast ultrasound image segmentation. Employing the BUSI and UDIAT Dataset B, HCMNet attained 96.82% accuracy, 70.96% IoU, and 83.01% DSC on the BUSI dataset and 98.93% accuracy, 80.06% IoU, and 88.82% DSC on the UDIAT Dataset B. The model improves segmentation precision while maintaining computational efficiency by including wavelet feature extraction and adaptive feature fusion.

Xiong et al. [24] presented MFPNet, a mixed feature perception network designed for skin lesion segmentation. This approach integrates global contextual attributes with local specifics to efficiently delineate lesion regions, employing a multi-scale feature perception module (MFPM) to improve resilience. MFPNet was evaluated on the ISIC2018 and PH2 datasets, attaining 96.19% accuracy, 83.84% IoU, and 91.21% DSC on ISIC2018 and 94.85% accuracy, 85.67% IoU, and 92.28% DSC on PH2.

### Critical Findings

Despite numerous studies in the field of breast cancer detection, there are still specific gaps that need to be addressed, particularly in the areas of data limitation and medical image feature engineering. Existing research has made significant strides in these areas; however, more refined techniques are necessary to enhance model robustness and generalizability further.

This study addresses these gaps by applying data augmentation techniques to balance the dataset and performing feature engineering by integrating multimodal features (radiomics and deep features) from the augmented training dataset. It enhances model learning while combining radiomics and deep learning-based features in a unified framework. We employed 13 transfer learning models, incorporating deep and extracted radiomics features, to assess their performance in early breast cancer detection. By integrating these techniques, our methodology enhances the feature extraction process and optimizes the training of the transfer learning models. This approach allows the models to benefit from both traditional medical image processing techniques (radiomics) and the powerful pattern recognition capabilities of deep learning, ultimately improving the model’s performance and robustness.

## 3. Materials and Methods

**Figure 1:**
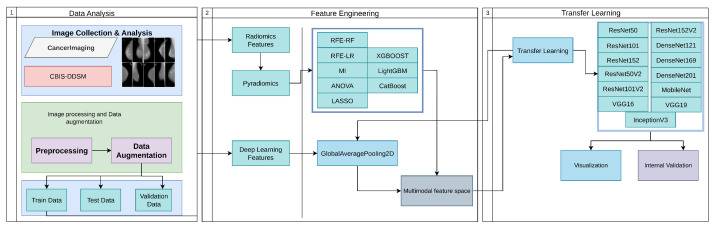
Workflow of the proposed methodology for breast cancer detection, comprising (1) data analysis, with image collection from CancerImaging and CBIS-DDSM datasets, preprocessing, and data augmentation; (2) feature engineering, integrating radiomics and deep learning features to form a multimodal feature space; and (3) transfer learning using models like ResNet50, ResNet152v2, DenseNet121, and others, followed by visualization and internal validation.

Our proposed approach organizes the research process into several distinct phases. Figure 1 describes our research methodology. The methodology commenced with the acquisition and analysis of breast imaging data. Image data were collected from the CBIS-DDSM [25] dataset and prepared through essential analysis steps. The data analysis phase included fixing image paths, determining the length of the dataset, mapping labels, counting the instances with respect to labels, and displaying image data. During the analysis, we identified an imbalanced dataset concerning labels (benign, malignant). Our next step involved applying image processing and data augmentation. In this step, we ensured that images were prepared to comply with the requirements of the pre-trained model and pyradiomics. We also carried out data augmentation to address the imbalanced dataset issue. After that, our augmented dataset was split into three subsets: training, validation, and testing, with a distribution ratio of 80% for training, 10% for validation, and 10% for testing. In the feature engineering phase, we employed two approaches: a radiomics features approach and a deep learning features approach. We extracted features for both approaches from our training data. Radiomics features were derived using the PyRadiomics library. For deep learning features, we utilized GlobalAveragePooling2D from pretrained models to extract high-level representations. To optimize the radiomics feature set, feature selection was performed using RFE (random forest, logistic regression), ANOVA F-test, LASSO, and mutual information criteria. In addition, embedded selection was explored with XGBoost, LightGBM, and CatBoost using GPU acceleration. The selected features were then combined with deep features to create a unified multimodal feature space for downstream model training. All features were standardized using z-score normalization after missing value imputation with a k-nearest neighbors imputer (*k* = 5). In the final phase, we implemented 13 transfer learning models for early breast cancer detection. We rigorously evaluated the model’s implementation using metrics such as accuracy, precision, recall, and F1 score, ensuring that it was accurate and reliable for early breast cancer detection. This structured approach, coupled with the rigorous assessment, enhances diagnostic precision and robustness, providing a solid foundation for accurate and actionable cancer predictions. Our proposed methodology has been implemented and can be accessed through the gitHub repository [26].

### 3.1. Dataset

**Figure 2:**
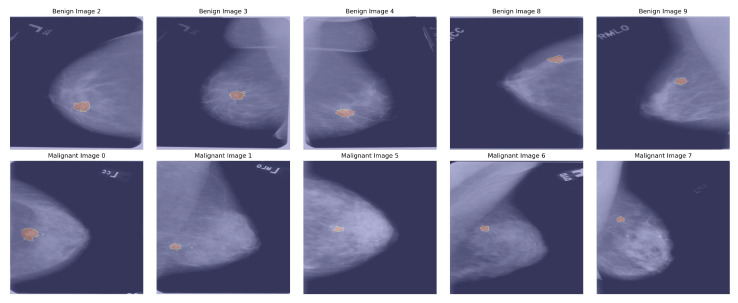
Mammogram images with highlighted regions showing segmented ROI.

We initially collected data from the well-known dataset CBIS-DDSM [25], which contains 2620 mammography images. This collection comprises 2620 mammography studies, encompassing normal, benign, and malignant cases. The dataset comprises updated ROI segmentation and bounding boxes, along with the pathological assessment for the training data. Figure 2 describes mammographic images with a segmented ROI.

### 3.2. Image Pre-Processing and Data Augmentation

After collecting data from the dataset, we applied several image processing techniques to ensure consistency and quality across the images. Algorithm 1 describes our image processing steps. First, we rescaled each image to a standardized resolution (224×224), ensuring uniformity and compatibility in subsequent feature extraction and model training stages.

(1) $$I^{\prime }\left(u,v\right)=\sum_{i=0}^{1}\sum_{j=0}^{1}w_{ij}\cdot I\left(x+i,y+j\right),$$

where

(2) $$x=\frac{u\cdot W}{M},\;y=\frac{v\cdot H}{N},$$

and W×H is the original image size, M×N=224×224 is the target size, and $w_{ij}$ are interpolation weights based on the relative distances to the four nearest neighboring pixels in the original image.
**Algorithm 1** Image Preprocessing Pipeline1:**procedure** PreprocessImage(*I*)2:      $I_{\mathrm{resized}}\leftarrow \mathrm{Rescale}\left(I,M,N\right)$3:      $I_{\mathrm{norm}}\leftarrow \mathrm{Normalize}\left(I_{\mathrm{resized}}\right)$4:      $I_{\mathrm{smooth}}\leftarrow \mathrm{GaussianFilter}\left(I_{\mathrm{norm}},\sigma \right)$5:      $I_{\mathrm{aligned}}\leftarrow \mathrm{RegisterImage}\left(I_{\mathrm{smooth}},\mathrm{reference}\right)$6:      **return** $I_{\mathrm{aligned}}$

We then performed normalization, standardizing pixel intensity values to reduce variations caused by lighting or contrast differences, allowing the model to focus on essential features rather than inconsistencies.

(3) $$I_{\mathrm{norm}}\left(u,v\right)=\frac{I^{\prime }\left(u,v\right)-\mu }{\sigma },$$

where μ and σ are the precomputed mean and standard deviation of pixel intensities across the dataset

To enhance clarity, we applied noise reduction using a Gaussian filter, minimizing background noise and artifacts that could interfere with accurate feature extraction.

(4) $$G\left(x,y\right)=\frac{1}{2\pi \sigma^{2}}\exp\left(-\frac{x^{2}+y^{2}}{2\sigma^{2}}\right),$$

and the denoised image $I_{\mathrm{smooth}}$ is obtained via convolution:

(5) $$I_{\mathrm{smooth}}\left(x,y\right)=\left(I_{\mathrm{norm}}*G\right)\left(x,y\right)=\sum_{i=-k}^{k}\sum_{j=-k}^{k}I_{\mathrm{norm}}\left(x-i,y-j\right)\cdot G\left(i,j\right),$$

where *k* is the kernel radius, typically chosen as k=⌈3σ⌉, ensuring sufficient coverage of the kernel’s support.

Finally, we used anatomical alignment to orient the images to a common reference point, ensuring that comparable regions are captured across images. Together, these processing steps refined the dataset, enabling reliable, high-quality input for analysis.

(6) $$\left[\begin{array}{c} x^{\prime }\\ y^{\prime } \end{array}\right]=\left[\begin{array}{cc} a_{11} & a_{12}\\ a_{21} & a_{22} \end{array}\right]\left[\begin{array}{c} x\\ y \end{array}\right]+\left[\begin{array}{c} t_{x}\\ t_{y} \end{array}\right]$$

We employed data augmentation techniques to mitigate the imbalance in the original dataset. Table 1 provides an overview of the CBIS-DDSM dataset, detailing the distribution of images across various labels. Each label includes a specific number of both original and augmented images. The augmentation pipeline (Algorithm 2) includes several transformations: random horizontal and vertical flips to simulate different orientations and random adjustments in brightness, contrast, saturation, and hue to mimic lighting and color variations. In addition, in the image processing stage, we applied a Gaussian filter to reduce background noise and artifacts to improve image clarity. Subsequently, during this phase, a Gaussian filter is applied with a random standard deviation to introduce blur effects to simulate real-world imaging variations, while random cropping followed by resizing simulates zoom-level variations. These strategies aim to mitigate the imbalance and enhance the model’s generalization capabilities. At the time of image data augmentation, we augmented 3 types of images that were collected from the dataset—full images, cropped images, and segmented ROI images. In our data analysis, we found that cropped and segmented ROI images are both related to full images. In Figure 2, we describe the full images with their corresponding segmented ROIs. Therefore, when applying augmentation techniques, we made sure to include all types of related images to maintain coherence in our dataset. After augmentation, a count of 8498 images per label was generated. The dataset is split into three subsets: training, validation, and testing, with a distribution ratio of 80% for training, 10% for validation, and 10% for testing. Due to mini-batches with a batch size of 32 and 64, the allocation of samples to each subset slightly deviated from the intended split. As a result, the size of the final datasets was as follows. The final datasets consist of 13600 training images (425 batches of 32 samples each), 1700 validation images (53 batches of 32 samples each), and 1700 testing images (53 batches of 32 samples each). The dataset was shuffled prior to splitting to ensure fairness and consistency, and the same random seed was utilized in each run. Furthermore, samples that could not form complete batches were excluded, minimizing potential bias in the dataset.
**Algorithm 2** Image augmentation pipeline**Require:** Image $I_{\mathrm{aligned}}$**Ensure:** Augmented Image $\hat{I}$   1:I←tf.image.random_flip_left_right(I)   2:I←tf.image.random_flip_up_down(I)   3:I←tf.image.random_brightness(I,0.3)   4:I←tf.image.random_contrast(I,0.8,1.2)   5:I←tf.image.random_saturation(I,0.8,1.2)   6:I←tf.image.random_hue(I,0.02)   7:σ←tf.random.uniform([],0.1,2.0)   8:I←tf.image.gaussian_filter2d(I,(3,3),σ)   9:s←tf.shape(I)[:2]×tf.random.uniform([],0.8,1.0) 10:I←tf.image.random_crop(I,s) 11:I←tf.image.resize(I,tf.shape(I)[:2]) 12:$I^{\prime }\leftarrow \mathrm{tf}.\mathrm{random}.\mathrm{normal}\left(\mathrm{tf}.\mathrm{shape}\left(I\right),0.0,0.05\right)$ 13:$I\leftarrow \mathrm{tf}.\mathrm{clip}\_\mathrm{by}\_\mathrm{value}(I+I^{\prime },0.0,1.0)$ 14:**return** Augmented Image $\hat{I}$

### 3.3. Feature Engineering

#### 3.3.1. Radiomics Features

In this phase, we conducted feature engineering, meticulously dividing it into feature extraction with radiomics feature selection and combination to create a robust feature set. During feature extraction, we gathered a comprehensive range of radiomic features from segmented regions of interest (ROIs). Algorithm 3 describes the radiomics extraction process. These features included first-order statistics (e.g., percentiles, energy, and entropy), shape-based descriptors (e.g., elongation, major axis length, and perimeter), and higher-order texture features derived from standard radiomics matrices, such as the gray-level run length matrix (GLRLM), gray-level size zone matrix (GLSZM), gray-level dependence matrix (GLDM), gray-level co-occurrence matrix (GLCM), and neighborhood gray-tone difference matrix (NGTDM). To ensure precision and consistency, we utilized the PyRadiomics library [27], which is specifically designed for extracting diverse radiomic features critical in medical imaging analysis. By combining radiomic features extracted through these methods, our approach ensures the integration of diverse quantitative descriptors, thereby creating a comprehensive feature set for analysis.
**Algorithm 3** Radiomics feature extraction**Require:** Dataframe $full_{m}ass$ with columns: patient_id, image_file_path, ROI_mask_file_path, labels**Ensure:** Radiomics features DataFrame $features_{d}f$   1:extractor←featureextractor.RadiomicsFeatureExtractor()   2:$extractor.enableFeatureClassByName{(}^{\prime }shape2D^{\prime })$   3:$features_{l}ist\leftarrow \left[\right]$   4:**for** each row in $full_{m}ass$ **do**   5:      $patient_{i}d,image_{p}ath,mask_{p}ath,level\leftarrow \mathrm{row}\;\mathrm{values}$   6:      $image\leftarrow \mathrm{cv}2.\mathrm{imread}(image_{p}ath,\mathrm{cv}2.\mathrm{IMREAD}\_\mathrm{GRAYSCALE})$   7:      $mask\leftarrow \mathrm{cv}2.\mathrm{imread}(mask_{p}ath,\mathrm{cv}2.\mathrm{IMREAD}\_\mathrm{GRAYSCALE})$   8:      **if** image.shape≠mask.shape **then**   9:           new_size←(image.shape[1],image.shape[0]) 10:           mask←cv2.resize(mask,new_size,cv2.INTER_NEAREST) 11:      $image_{s}itk\leftarrow \mathrm{sitk}.\mathrm{GetImageFromArray}\left(image\right)$ 12:      $mask_{s}itk\leftarrow \mathrm{sitk}.\mathrm{GetImageFromArray}\left(mask\right)$ 13:      $result\leftarrow extractor.execute(image_{s}itk,mask_{s}itk)$ 14:      $features_{l}ist.append\left(\mathrm{result}{,}^{\prime }patient_{i}d^{\prime }:patient_{i}d{,}^{\prime }labels^{\prime }:level\right)$ 15:$features_{d}f\leftarrow \mathrm{pd}.\mathrm{DataFrame}\left(features_{l}ist\right)$ 16:$\mathrm{features}\_df.\mathrm{to}\_\mathrm{csv}{(}^{\prime }radiomics_{f}eatures.csv^{\prime },\mathrm{index}=\mathrm{False})$ 17:**return** $features_{d}f$

#### 3.3.2. Radiomics Feature Selection

Feature selection is critical for radiomics features due to the high-dimensional nature of radiomics data. This high dimensionality can lead to overfitting, where models perform well on training data but fail to generalize to new data, especially given the often limited sample sizes in medical studies. Feature selection mitigates the curse of dimensionality by reducing the feature space, improving model performance, robustness, and generalizability. In our study, we systematically compared a broad range of supervised feature selection techniques with varying subset sizes. Recursive feature elimination (RFE) was applied using random forest and logistic regression base classifiers, selecting 10, 20, 50, and 100 features. In addition, RFECV automatically determined the optimal number of features, resulting in 74 for random forest and 647 for logistic regression. Univariate feature selection based on the ANOVA F-statistic was performed via SelectKBest with 10, 20, 50, and 100 features. LASSO (LassoCV) was applied, yielding 90 and 157 selected features on a fivefold cross-validation configuration. Mutual Information feature ranking was also evaluated, retaining the top 50, 100, and 200 features. Embedded methods using tree-based models with GPU acceleration were explored through XGBoost, LightGBM, and CatBoost, with feature subset sizes of 50, 100, and 200. The methods and their corresponding number of selected features are summarized in Table 2.

These diverse feature selection pipelines aimed to identify the most discriminative and robust subsets, which were subsequently integrated with deep features to build a unified multimodal feature space for final model development.

#### 3.3.3. Deep Learning Features

In addition to radiomics features, we utilized GlobalAveragePooling2D for deep learning feature extraction. GlobalAveragePooling2D is an efficient method for transitioning from convolutional layers to fully connected layers, enabling effective feature extraction while reducing the risk of overfitting. This operation condenses the spatial dimensions of the feature maps by computing the average of all spatial positions for each feature map channel. Mathematically, for a feature map Fm with dimensions J×E×X, where *J* = height, *E* = width, and *X* = number of channels, the global average pooling operation computes the output for the *c*-th channel as:

(7) $$f_{c}=\frac{1}{J\times E}\sum_{k=1}^{J}\sum_{l=1}^{E}Fm_{k,l,c}$$

where

- $F_{k,l,c}$ = activation value at position (k,l) in the *c*-th feature map.
- $f_{c}$ = resultant scalar for the *c*-th channel after applying the global average pooling.

This operation ensures that each channel contributes a single value to the final feature vector, significantly reducing the dimensionality while retaining global spatial information. By summarizing the feature maps into compact vectors, GlobalAveragePooling2D enhances the model’s generalization ability and reduces the risk of overfitting. The extracted deep features are combined with radiomics features to form a comprehensive input representation, enabling the model to achieve high classification accuracy.

### 3.4. Multimodal Feature Preparation

After independently processing both radiomics and deep features, we applied several data harmonization steps to prepare them for fusion and downstream machine learning. First, missing values in the radiomics features are addressed using a KNN imputer with *k* = 5, which leverages neighboring samples to estimate missing entries. The same KNN imputer is also applied to the deep features to handle potential missing activations that could occur if, for instance, specific images failed to process fully. Algorithm 4 describes the multimodal feature preparation.

Once imputed, both radiomics and deep features undergo standard normalization using a StandardScaler (z-score standardization) to ensure comparable scales across heterogeneous features. This is crucial because radiomics features (often with units such as pixel intensity) and deep features (unitless activation patterns) can have substantially different distributions otherwise.
**Algorithm 4** Multimodal feature preparation**Require:** Radiomics feature matrix *R*, deep feature matrix *D*, target labels *y***Ensure:** Preprocessed and fused feature matrix *F*   1:Apply KNN imputation with k=5 to *R*, producing $R_{imp}$   2:Apply KNN imputation with k=5 to *D*, producing $D_{imp}$   3:Standardize $R_{imp}$ with zero mean and unit variance, yielding $R_{norm}$   4:Standardize $D_{imp}$ with zero mean and unit variance, yielding $D_{norm}$   5:Rename columns of $D_{norm}$ as deep_feature_i for traceability   6:Concatenate $R_{norm}$ and $D_{norm}$ horizontally to form *F*   7:Align *F* rows to patient IDs from *y*   8:**return** *F*

### 3.5. Transfer Learning Models

The experiment setup, as detailed in Table 3, outlines the architectural and training configuration applied across 13 different transfer learning models. Every model was set up with weights that were pre-trained on ImageNet to leverage hierarchical features for the CBIS_DDSM dataset with 2 classes, and the dimensions of the input image were specified as (224, 224, 3); for InceptionV3, it was (299, 299, 3) to reflect its multi-scale inception modules. Architectural choices were task-driven: VGG16’s 13 convolutional layers with 3 × 3 kernels ensured simplicity, while ResNet50’s 50 layers, with 7 × 7, 1 × 1, and 3 × 3 kernels, enabled robust feature extraction via bottleneck blocks. To fine-tune the models, the top 30% of layers were unfrozen while keeping the remaining layers frozen to prevent overfitting and catastrophic forgetting. Regularization techniques included L2 weight decay with λ=0.01 and dropout layers with 30% applied to dense layers, minimizing overfitting by encouraging simpler models and robust feature representations. The categorical cross-entropy loss function was used, with the Adam optimizer configured at a learning rate of $1\times 10^{-5}$, which is ideal for multi-class classification, as it measures the difference between predicted probabilities and one-hot encoded labels, encouraging the model to assign high probabilities to the correct class, thus optimizing classification accuracy. The low learning rate of $1\times 10^{-5}$ is critical for fine-tuning, as it enables precise weight updates to the top 30% of unfrozen layers, preserving pretrained features while adapting to the new task, thus preventing overfitting and catastrophic forgetting. Early stopping with a patience value of 5 epochs was implemented to halt training when validation performance stops improving, preventing overfitting and saving computational resources, and the best model for each architecture was saved in .h5 format. The models’ performance was analyzed using accuracy/loss curves, confusion matrices, and classification reports to assess their effectiveness in classification tasks.

**Figure 3:**
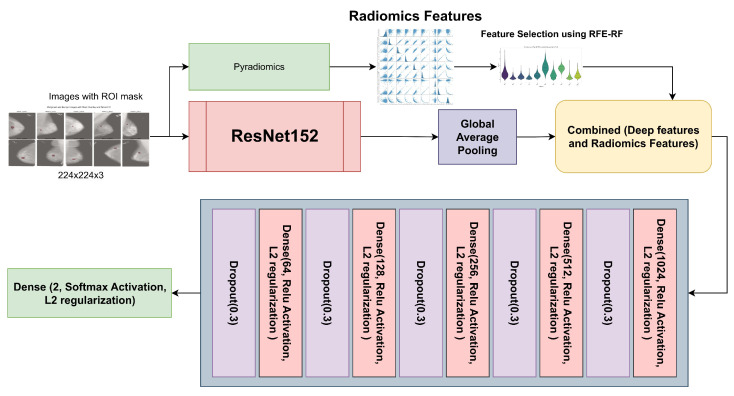
ResNet152 with radiomics and custom input for breast cancer classification.

We achieved the highest accuracy with the ResNet152 model. We utilized the ResNet152 architecture with custom layers to integrate radiomics features for improved performance in medical image classification. Figure 3 outlines the structure of the ResNet152 model combined with custom layers. The base model, ResNet152, is a deep convolutional neural network that begins with an initial convolutional layer using a 7 × 7 kernel, 64 filters, and a stride of 2, followed by max-pooling and several residual blocks. The residual blocks are structured with multiple convolutional layers featuring progressively increasing filter sizes of 128, 256, 512, and 1024. This architecture enhances the network’s capability to capture features at both low and high levels accurately, thus alleviating the vanishing gradient issue and strengthening the model’s capacity to recognize intricate patterns. After the residual blocks, global average pooling effectively compresses the feature maps into a 1D vector. To further enhance the model, custom layers integrate radiomics features into the network. The deep features from ResNet152 and the radiomics features are concatenated, followed by several fully connected layers with ReLU activation, L2 regularization, and dropout for regularization and to prevent overfitting. For binary classification in the final output layer, we utilized a Softmax activation function, such as distinguishing between benign and malignant conditions. This combined architecture effectively integrates deep learning features and radiomics information, improving the model’s overall classification performance.

### 3.6. Model Evaluation

We established an internal validation procedure with an 80%, 10%, and 10% division for training, validation, and testing. This method assured that class distributions were maintained throughout all subsets. We evaluated the model’s effectiveness using metrics including accuracy, precision, recall, specificity, F1 score, and area under the curve.

Accuracy (Ac):

(8) $$\mathrm{Ac}=\frac{\mathrm{TP}+\mathrm{TN}}{\mathrm{TP}+\mathrm{TN}+\mathrm{FP}+\mathrm{FN}}$$

Precision (Prec):

(9) $$\mathrm{Prec}=\frac{\mathrm{TP}}{\mathrm{TP}+\mathrm{FP}}$$

Recall (Reca):

(10) $$\mathrm{Reca}=\frac{\mathrm{TP}}{\mathrm{TP}+\mathrm{FN}}$$

Specificity (Spec):

(11) $$\mathrm{Spec}=\frac{\mathrm{TN}}{\mathrm{TN}+\mathrm{FP}}$$

F1 score (F1s):

(12) $$F1s=2\times \frac{\mathrm{Prec}\times \mathrm{Rec}}{\mathrm{Pre}+\mathrm{Rec}}$$

Area under the curve (AUC):

(13) $$\mathrm{AUC}=\int_{-\infty }^{\infty }\mathrm{TPR}\left(t\right)\;d\mathrm{FPR}\left(t\right)$$

## 4. Results

### 4.1. Radiomics Analysis

In our radiomics analysis model, we extracted seven types of radiomic features using pyradiomics. Table 4 describes the radiomics features by categories. A total of 1040 radiomic features were extracted from the imaging dataset. Among these, first-order statistical features accounted for 198 features, capturing voxel intensity distributions within the region of interest. Texture features were highly represented, including 264 features from the gray-level co-occurrence matrix (GLCM), 176 from the gray-level run length matrix (GLRLM), 176 from the gray-level size zone matrix (GLSZM), 154 from the gray-level dependence matrix (GLDM), and 55 from the neighborhood gray-tone difference matrix (NGTDM). Additionally, nine shape-based features were included to characterize lesion geometry, along with five diagnostic metadata features.

#### Radiomics Feature Selection

Our study evaluates feature selection methods across five categories (RFE, SelectKBest, LASSO, tree-based, and information-based) with different numbers of features. Table 5 describes the comprehensive comparison of radiomics feature selection methods. In our study, we use metrics such as accuracy, F1 score, precision, recall, and stability. Our analysis reveals that recursive feature elimination (RFE) with random forest (n = 100) achieves the highest accuracy (83.7%), F1 score (83%), precision (86.2%), recall (80.0%), and moderate stability (0.485). SelectKBest (ANOVA, n = 20) offers the highest stability (0.897). No significant performance differences were found across method categories (ANOVA *p* = 0.699). Methods achieving high performance (F1 >0.82) with fewer features (≤20) suggest significant feature redundancy in radiomics data.

**Figure 4:**
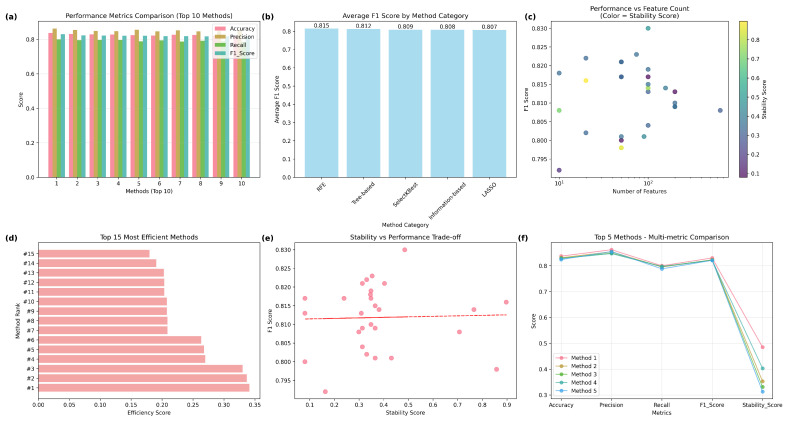
Feature selection comprehensive analysis, (**a**) Performance Metrics Comparison (Top 10 Methods); (**b**) Average F1 Score by Method Category comparing F1 scores across categories; (**c**) Performance vs Feature Count (Color = Stability Score) illustrating the relationship between the number of features and performance; (**d**) Top 15 Most Efficient Methods ranked by efficiency score; (**e**) Stability vs. Performance Trade-off plotting stability score against F1-score to show the trade-off relationship; (**f**) Multi-metric Comparison comparing accuracy, precision, recall, F1-score, and stability score across the top five methods.

Figure 4 summarizes the comparative analysis. Figure 4a compares accuracy, precision, recall, and F1 score across the top 10 performing methods. The best overall feature selection method was RFE with random forest using 100 features, achieving an F1 score of 83% and a stability score of 0.485, striking a balance between predictive performance and feature stability. In contrast, the SelectKBest method with ANOVA (n = 20) attained the highest stability (0.897), with a competitive F1 score (81.6%), highlighting its suitability for reproducible feature selection in clinical applications. Figure 4b shows average F1 scores by method category, showing RFE with the highest mean F1 score (81.5%), followed closely by the tree-based, SelectKBest, information-based, and LASSO methods, suggesting minimal differences in overall category-level performance. In a scatterplot, Figure 4c depicts the relationship between the number of selected features (log scale) and F1 score, with marker color indicating stability scores. This reveals that high F1 scores can be obtained with a broad range of feature counts, while stability is more variable, highlighting the trade-offs among model complexity, performance, and robustness. The horizontal bar chart in Figure 4d ranks the top 15 most efficient methods based on efficiency score (F1 score per feature count). This emphasizes the advantage of the RFE and SelectKBest methods in achieving competitive performance with relatively few features. The scatterplot in Figure 4e illustrates the F1 score in relation to the stability score, along with a trend line that reveals a near-zero correlation (Pearson r = 0.03), suggesting that stability and performance are predominantly independent, warranting the reporting of both in radiomics pipelines. Figure 4f shows a line plot comparing the top five methods across multiple metrics, demonstrating consistently high accuracy, precision, recall, and F1 scores but more variation in stability scores, highlighting differences in robustness among the top-performing methods.

The RFE-RF approach utilising 100 features attained the highest overall F1 score of 83% among all assessed methods, signifying exceptional classification performance. This approach utilizes the advantages of random forest, an ensemble technique known for its ability to capture intricate, nonlinear correlations in radiomics data, while systematically removing less useful elements. By selecting 100 features, it attains an effective balance between model complexity and overfitting mitigation, preserving generalizability on novel data. Furthermore, its moderate stability score (0.485) indicates that the chosen features exhibited reasonable consistency across cross-validation folds, instilling trust in their reproducibility while maintaining a focus on high prediction accuracy. In comparison to techniques exhibiting greater stability but inferior F1 scores, RFE-RF (n = 100) provides an ideal balance between efficacy and resilience.

### 4.2. Model Performance

Table 6 provides a comprehensive comparison of 13 transfer learning models, including DenseNet, InceptionV3, MobileNet, ResNet, and VGG, with a focus on key performance metrics: accuracy (Ac), precision (Prec), recall (Reca), specificity (Spec), F1 score (F1s), area under the curve (AUC), and the number of epochs (Ep) used for training. ResNet152 is the highest-performing model, achieving the best values across all metrics: a precision of 97%, recall of 98%, specificity of 96%, F1 score of 97%, AUC of 99.30, and accuracy of 97%. This makes ResNet152 particularly suitable for tasks that require a strong balance between minimizing false positives and accurately detecting true positives. VGG19 follows closely behind, with a precision, recall, specificity, F1 score, and accuracy of 96% and an AUC of 99%, confirming its high reliability in both precision and recall. Other models, such as DenseNet121, DenseNet169, and DenseNet201, exhibit solid performance but fall slightly short compared to ResNet152 and VGG19. These models show precision and recall values ranging from 93% to 95%, specificity values ranging from 95% to 96%, F1 scores of approximately 94%, AUC values ranging from 98.6% to 98.9%, and accuracy between 93% and 95%, making them practical for general use but less optimal than the top performers. InceptionV3 and MobileNet are relatively weaker performers in precision and recall, with InceptionV3 achieving an accuracy of 89% and MobileNet reaching 88%. These results suggest that these models are less reliable at correctly identifying positive instances. While InceptionV3 performs better than MobileNet, both models still lag behind the other architectures in precision and recall metrics. The ResNet50 and ResNet50V2 models exhibit consistent performance, with precision and recall values of approximately 94%, F1 scores of 94%, AUC values of 98.5% to 98.8%, and accuracy of 94%, positioning them as solid but not top-tier options. Models from the ResNet101 family, specifically ResNet101 and ResNet101V2, perform at a high level, with precision, recall, and F1 scores of 96% and an AUC of 99%, along with an accuracy of 96%, indicating their robustness for tasks requiring strong model performance across all metrics. Notably, the ResNet152 model requires fewer epochs (40 epochs) than other models, such as ResNet101 and ResNet50, which were trained for 50 epochs, highlighting its more efficient convergence. These results demonstrate that while ResNet152 and VGG19 dominate overall performance, models like DenseNet and ResNet101 provide strong alternatives. Some offer faster convergence with minimal sacrifice in accuracy.

**Figure 5:**
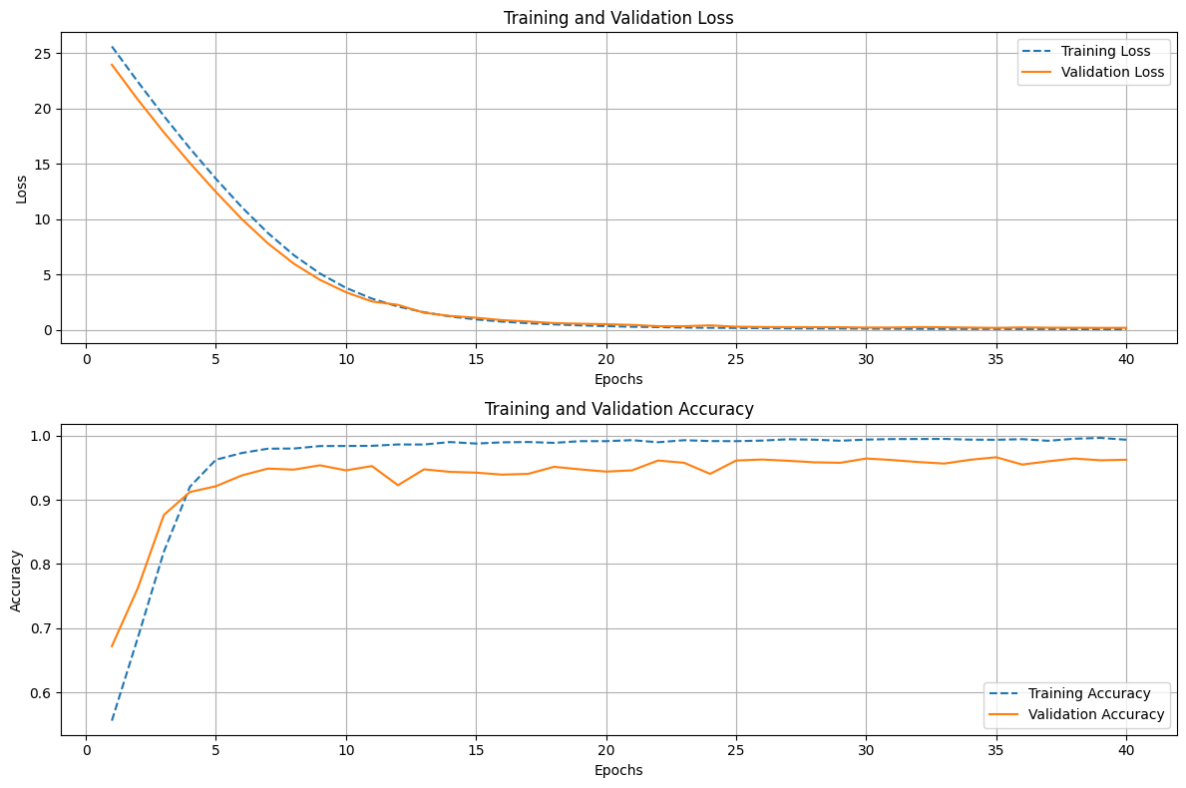
Training and validation loss (**top**) and accuracy (**bottom**) of ResNet152 over 40 epochs. Loss decreases sharply before stabilizing, while accuracy rises quickly and remains high, indicating effective learning and generalization.

**Figure 6:**
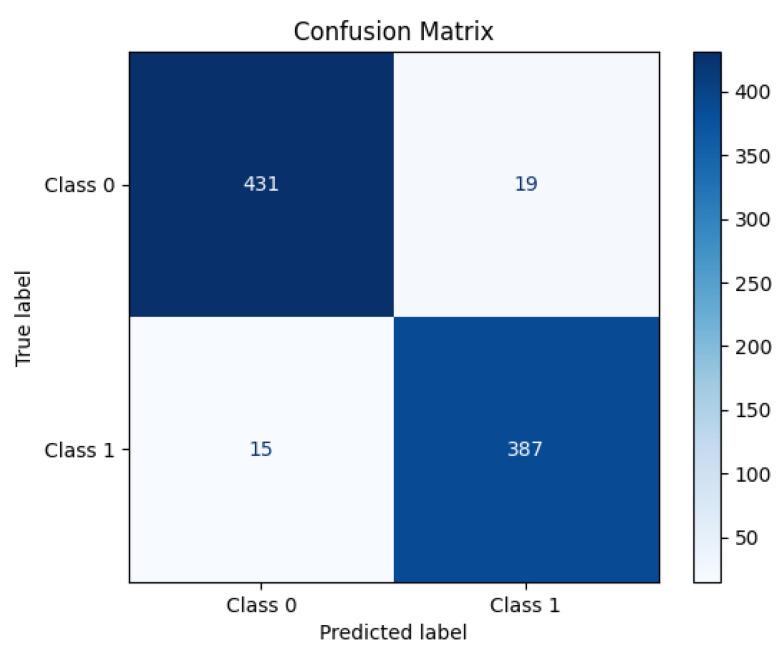
Confusion matrix for ResNet152 showing classification performance with minimal misclassifications and high accuracy.

Figure 5 shows the training and validation performance of the ResNet152 model over 40 epochs. The upper graph displays both the training and validation loss. The training loss decreases sharply in the initial epochs, indicating that the model is quickly learning to minimize errors. The loss gradually converges as training progresses, reflecting stability in the learning process. The validation loss follows a similar trend, steadily decreasing before leveling off, suggesting that the model generalizes well to unseen data. The lower graph illustrates the accuracy of training and validation. During the early epochs, we see a rapid increase in training accuracy, approaching near-perfect classification. The validation accuracy also trends upward but displays minor fluctuations, which is typical due to variations in the validation dataset. The consistent gap between training and validation accuracy remains small, indicating that the model does not suffer from significant overfitting. The ResNet152 model achieves high accuracy with stable convergence, demonstrating its strong learning capacity and generalization performance. Figure 6 presents the confusion matrix for the ResNet152 model, illustrating its classification performance. The model correctly classified 431 instances of Class 0 (TN) and 387 instances of Class 1 (TP), demonstrating high accuracy. However, 19 samples of Class 0 were misclassified as Class 1 (FP), and 15 samples of Class 1 were incorrectly predicted as Class 0 (FN). These misclassifications indicate a small prediction error, though the model maintains strong classification performance overall. The high concentration of values along the diagonal of the matrix suggests that ResNet152 effectively distinguishes between the two classes, achieving a robust balance between precision and recall.

**Figure 7:**
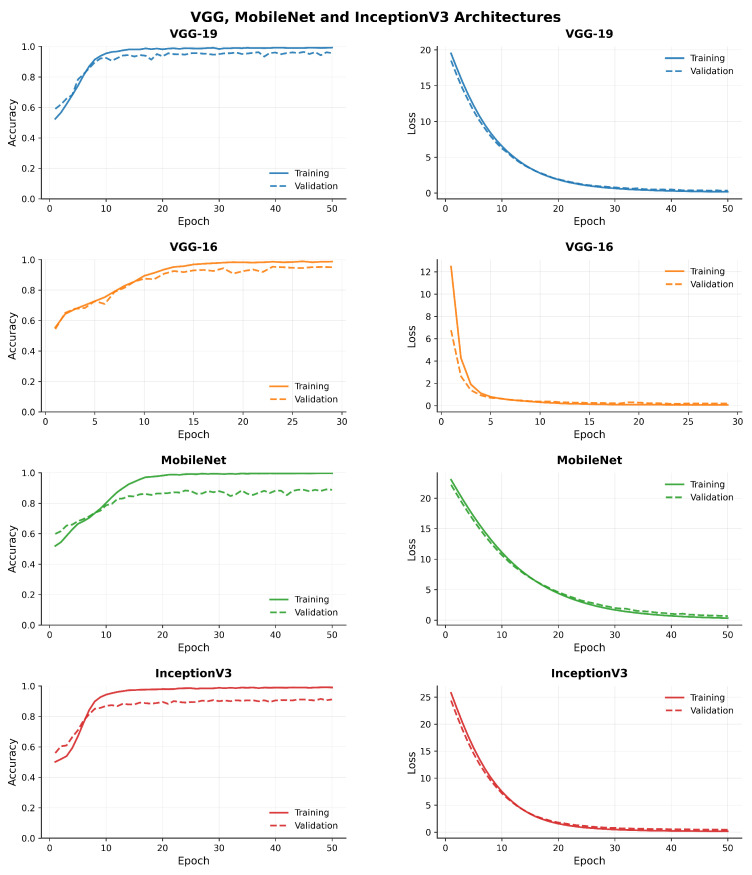
Comparison of model accuracy and loss across transfer learning (VGG, MobileNet, and InceptionV3) architectures.

**Figure 8:**
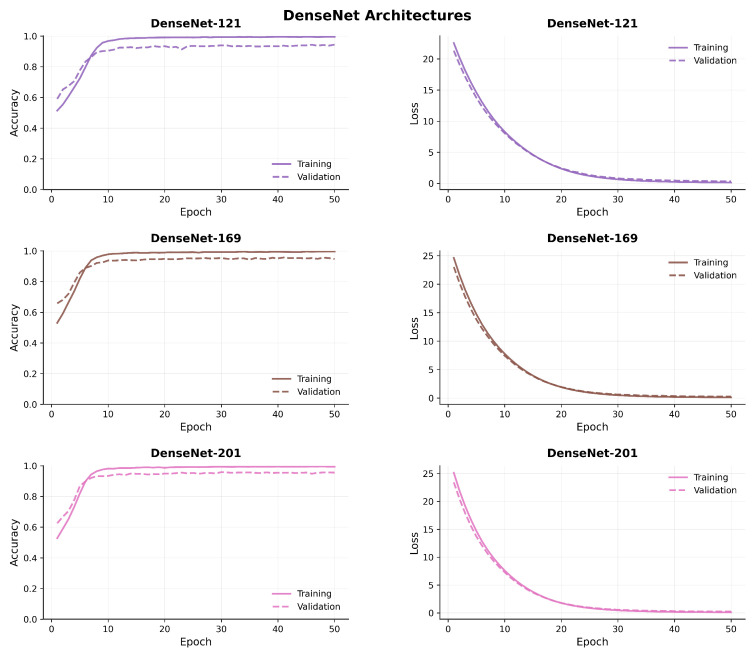
Comparison of model accuracy and loss across transfer learning (DenseNet) architectures.

**Figure 9:**
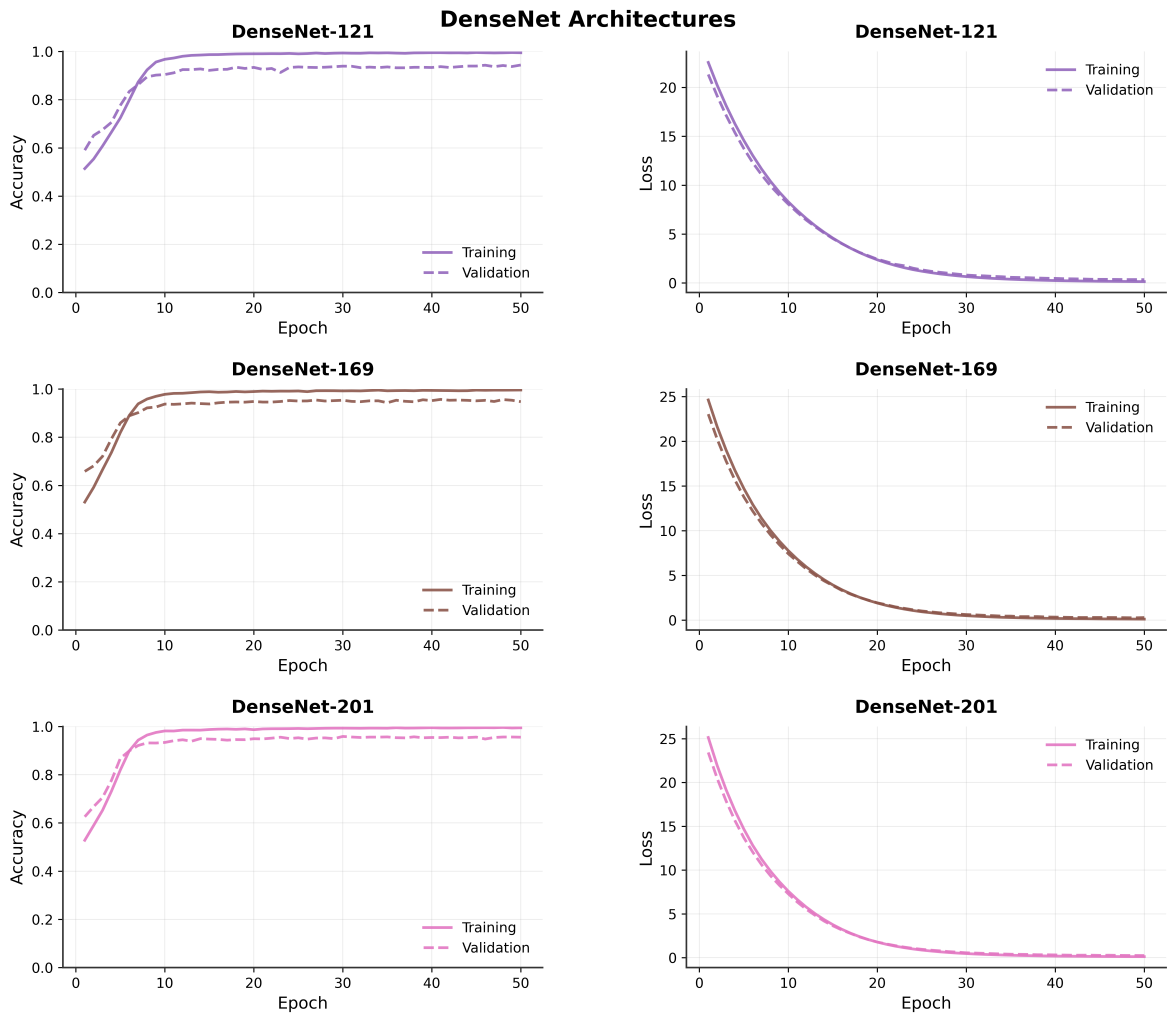
Comparison of model accuracy and loss across transfer learning (ResNet 50, 101, and 152) architectures.

**Figure 10:**
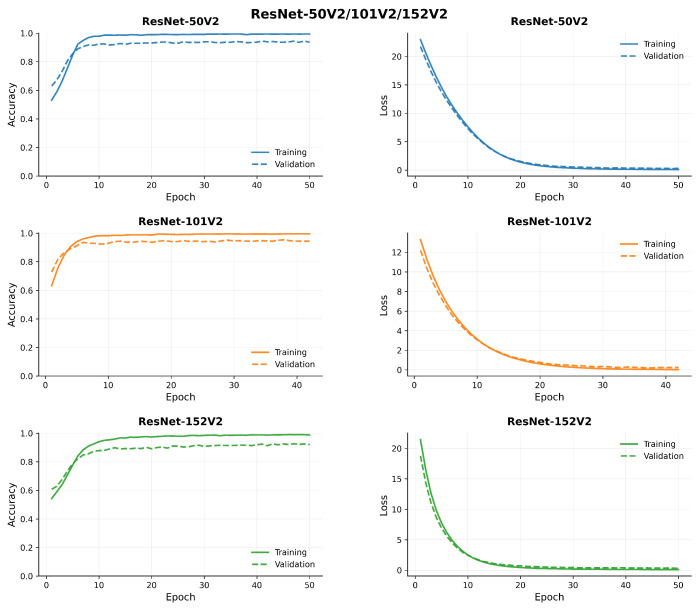
Comparison of model accuracy and loss across transfer learning (ResNet 50V2, 101V2, and 152V2) architectures.

Figure 7, Figure 8, Figure 9 and Figure 10 compare model accuracy and loss over 50 epochs. Training and validation accuracy are plotted on the left, showing the performance improvement of each model as training progresses, with higher accuracy indicating better performance. Training and validation loss are plotted on the right, reflecting that the models minimize error during training; lower loss values suggest better optimization. The models’ accuracy increases while loss decreases, highlighting their learning and generalization capabilities.

**Figure 11:**
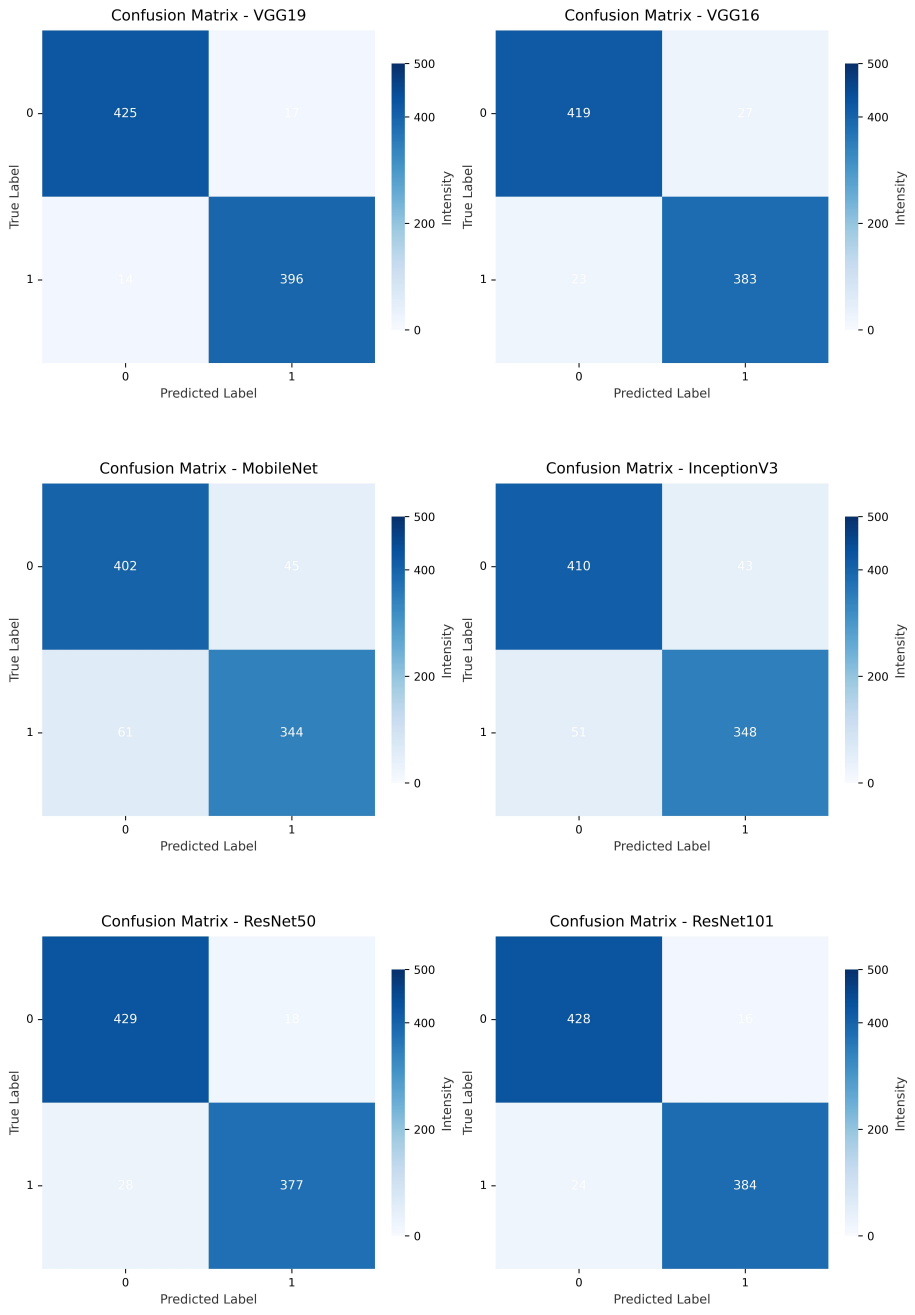
Comparison of confusion matrix across transfer learning architectures (VGG16, 19, MobileNet, InceptionV3, ResNet50, 101).

**Figure 12:**
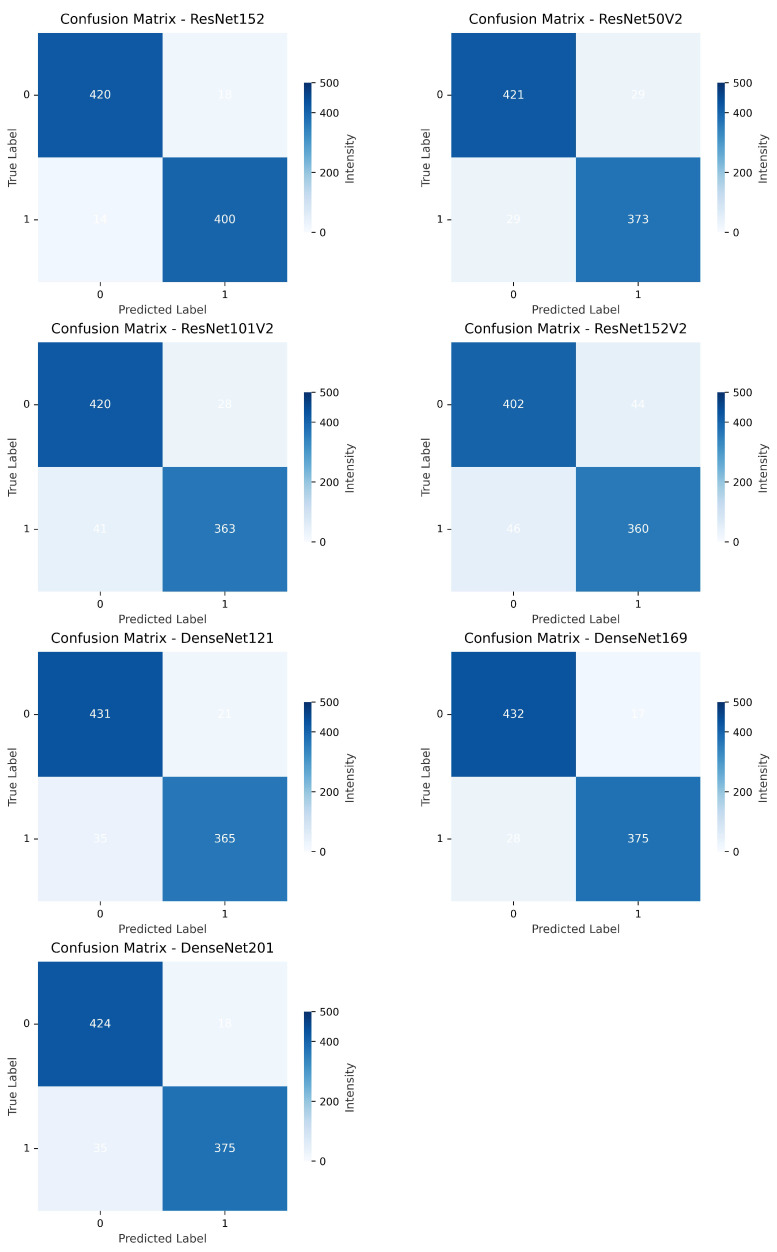
Comparison of confusion matrices across transfer learning architectures (ResNet152, (50, 101, 152) V2, DenseNet (121, 169, 201)).

Figure 11 and Figure 12 compare the confusion matrices of transfer learning models. The correct prediction range for all models is 396 to 433 for Class 0 and 334 to 400 for Class 1. Misclassifications vary from 14 to 61 for false positives and 17 to 51 for false negatives. Models such as VGG19, ResNet50, and DenseNet201 exhibit higher accuracy with counts like 425 and 375, whereas MobileNet and DenseNet169 show more errors, with 61 and 28 misclassifications, respectively, providing a clear comparison of model performance.

**Figure 13:**
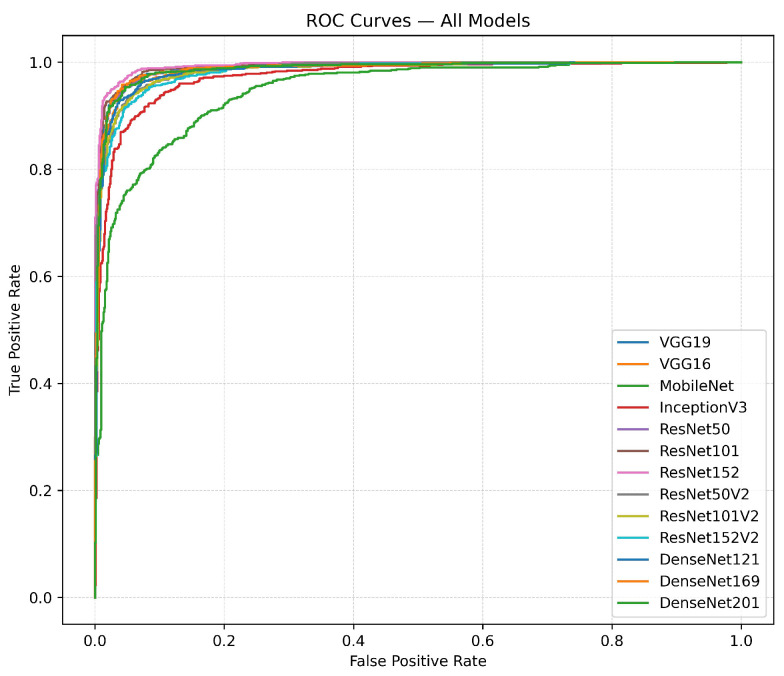
The ROC curves highlight the performance of transfer learning models, with ResNet152, VGG19, and DenseNet169, showing the strongest balance between true-positive and false-positive rates.

**Figure 14:**
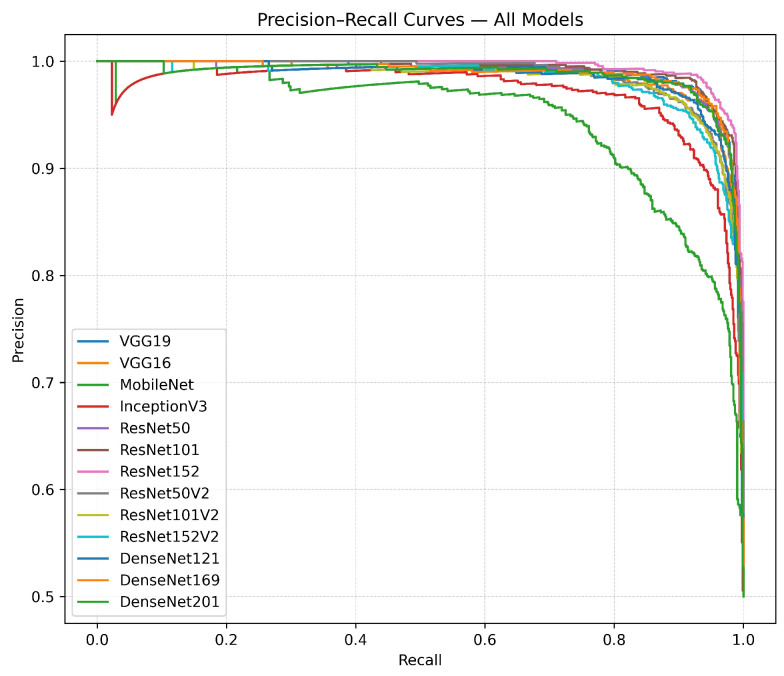
The precision–recall curves illustrate the trade-off between precision and recall, with ResNet152 and VGG19 maintaining high precision across a wide recall range, outperforming models like MobileNet and InceptionV3.

Figure 13 and Figure 14 present the ROC curves and precision–recall (PRC) curves for the transfer learning models. The ROC curves illustrate the relationship between the true-positive rate (TPR) and the false-positive rate (FPR) across various models. Models like ResNet152, VGG19, and DenseNet169 exhibit the most significant initial increases, sustaining high true-positive rates despite rising false-positive rates, indicating their exceptional discriminative capabilities. In comparison, MobileNet and InceptionV3 show more gradual curves, suggesting less effective performance in differentiating between classes. The precision–recall curves provide additional understanding by graphing precision in relation to recall. The performance of ResNet152 and VGG19 is characterized by curves that maintain high precision over a broad spectrum of recall values, indicating strong effectiveness across different thresholds. DenseNet169 stands out with a consistently high performance curve, maintaining its elevated position in comparison to less effective models such as MobileNet and InceptionV3, which exhibit more pronounced drops in precision as recall rises.

### 4.3. Model Performance Statistical Analysis

**Figure 15:**
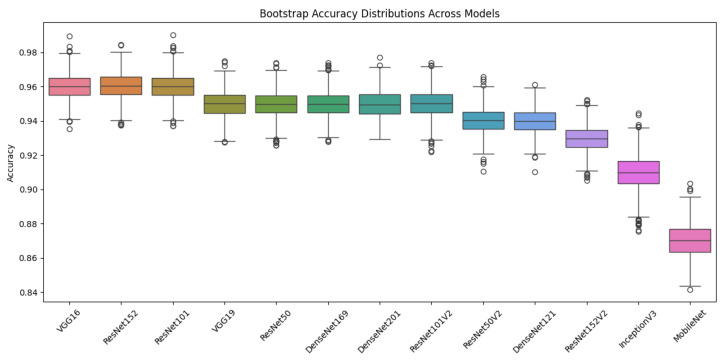
Bootstrap accuracy distributions across models. The box plot shows the distribution of accuracies obtained through bootstrapping, allowing comparison of model performance variability.

**Figure 16:**
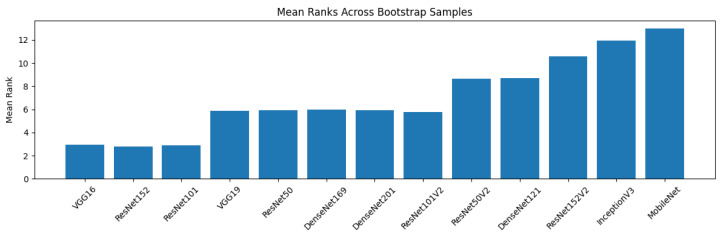
Mean ranks across bootstrap samples. A simplified critical difference diagram illustrates the average model ranks based on the Friedman test, allowing visual comparison of performance differences.

We analyzed the performance of 13 transfer learning models in classifying breast lesions using the CBIS-DDSM dataset. Performance metrics, including accuracy, F1 score, precision, and recall, are reported with 95% bootstrap confidence intervals (CIs) to reflect uncertainty (Figure 15 and Figure 16). As shown in Table 7, VGG16 and ResNet152 achieved the highest performance, each with an accuracy of 96% (95% CI: 94–97%) and an F1 score of 96% (95% CI: 94–97%). They are closely followed by ResNet101, VGG19, ResNet50, and several DenseNet models, with accuracies ranging from 95% to 96% and overlapping CIs. Lower-performing models, such as MobileNet, recorded an accuracy of 87% (95% CI: 85–89%). Top-performing models (VGG16, ResNet152, ResNet101, VGG19, ResNet50, DenseNet169, DenseNet201, and ResNet101V2) demonstrated strong cross-metric performance, suggesting robust generalization. ResNet50V2 and DenseNet121 showed slightly lower performance C, followed by ResNet152V2, InceptionV3, and MobileNet.

### 4.4. Statistical Comparison of Top Models

In order to analyze the differences between top-performing models, we conducted a paired statistical test on ResNet152 and VGG16. A two one-sided test (TOST) equivalence test with a margin of ±0.02 had a combined *p*-value of 0.000, meaning VGG16 and ResNet152 are equivalent in accuracy within the ±0.02 margin. Furthermore, McNemar’s test of per-sample prediction correctness gave a statistic of 12.552 with a *p*-value of 0.000. The findings revealed substantial differences in classification errors, which may be attributed to specific sample-based errors. These results indicate that while VGG16 and ResNet152 have equivalent overall performance, they may differ in specific failure modes. Further analysis of error overlap is needed to understand these differences.

### 4.5. Per-Class Performance

To evaluate clinical relevance, we analyzed per-class performance for the malignant class (Table 8). Both VGG16 and ResNet152 achieved high recall (95–96%) but low precision (29.3), indicating a high false-positive rate.

### 4.6. Post-Hoc Multiple Comparisons via Nemenyi Test

We used a nonparametric Friedman test and a subsequent Nemenyi post-hoc test to analyze all 13 models at the same time. For the Friedman test, the statistic was 8907.405, and the *p*-value was 0.000, indicating that the relative performance ranks given to the models by the different test datasets used were not the same between models, which suggested that their ranks were significantly different; thus, the models performed differently. Applying the Nemenyi test, we found that the best-performing models, which included VGG16, ResNet152, and ResNet101, were found not to differ significantly from each other (p estimate 1.0); however, they significantly surpassed MobileNet and InceptionV3 (*p* < 0.05). In conclusion, these findings indicate architectural differences among models; however, top-performing models generalize well on CBIS-DDSM. The significant differences between top and bottom models highlight the limitations of lighter architectures like MobileNet for this task.

### 4.7. Performance Comparison

We compare our results with those of recent studies published between 2022 and 2025. All of these studies utilized transfer learning models for evaluation. Table 9 presents a comparison of our research performance with these recent studies. Our study demonstrates superior performance across multiple deep learning architectures compared to the majority of the referenced studies. Notably, our implementation of ResNet-152 achieves the highest accuracy of 97%, surpassing all other models in the comparison. This is followed closely by our ResNet101 and ResNet101V2 models, both of which achieve 96% accuracy, as well as our VGG19 model, which also achieves 96%. These results indicate a significant improvement over earlier studies, such as Yu et al. [28], where the best-performing model (ResNet50) achieved 82% accuracy, and Wei et al. [29], where VGG19 reached 83%. Among the compared studies, Alexandru et al. [19] achieved 99.6% accuracy with DenseNet121, Sharmin et al. [30] and Wang et al. [22] reported a competitive accuracy of 95% and 96.4% using ResNet50V2 and custom CNN, which is closely matched by our ResNet101 (96%) and exceeded by our other ResNet architectures (ResNet101V2 and ResNet152). The performance of our ResNet50 (94%) also significantly outperforms the ResNet50 implementations by Yu et al. [28] (82%) and Wei et al. [29] (72%), highlighting the effectiveness of our transfer learning and optimization strategies. For VGG-based models, our VGG16 and VGG19 implementations achieve 94% and 96% accuracy, respectively, compared to 71% (VGG16, Yu et al. [28]) and 83% (VGG19, Wei et al. [29]). This substantial improvement underscores the robustness of our preprocessing and training methodologies. Similarly, our Inception_v3 model (89%) outperforms the Inception_v3 implementation by Wei et al. [29] (72%), although it is the least competitive among our models. Compared to Gao et al. [31] and Yang et al. [32], whose ResNet and 3DResNet models achieved 82% and 74% accuracy, respectively, our models consistently demonstrate higher performance across all tested architectures.

## 5. Discussion

In our study, after comprehensive data augmentation and feature engineering, we implemented 13 transfer learning models for early breast cancer detection.

Our evaluation of 13 transfer learning models for breast cancer detection offers helpful information about the relative effectiveness of deep neural network architectures in mammographic image analysis. ResNet152 achieved the highest accuracy (97%), followed closely by ResNet101, ResNet101V2 (96%), and ResNet50 (94%). This consistent superiority of the ResNet family underscores the benefit of residual connections in modeling complex hierarchical patterns in medical images, aligning with prior studies in radiological AI.

Notably, VGG19 also achieved competitive performance (96% accuracy), demonstrating that well-optimized, established architectures can remain viable alternatives, particularly in resource-constrained settings. While VGG models typically demand more memory during training, their simpler inference requirements may offer practical advantages in clinical deployment.

Statistical analysis further revealed nuanced differences among top-performing models. Although the TOST equivalence test confirmed that ResNet152 and VGG16 were equivalent within a ±0.02% accuracy margin (*p* < 0.001), McNemar’s test indicated significant differences in their prediction patterns $\chi^{2}=12.552$, p<0.001. This suggests divergent error profiles and complementary decision boundaries, supporting the potential of ensemble strategies to improve robustness and reduce model-specific biases.

The Friedman test revealed significant stratification in model performance: $\chi^{2}\left(12\right)=89.7$, *p* < 0.001, with post-hoc Nemenyi analysis confirming that lightweight architectures such as MobileNet and InceptionV3 underperformed compared to deeper models. This highlights the importance of architectural capacity for capturing subtle pathological features in mammography, challenging the assumption that efficiency-oriented models can match the performance of deeper networks in this domain.

The integration of deep features with radiomics features suggests effectiveness in creating a comprehensive image representation. Recursive feature elimination (RFE) with random forest yielded the best F1 score (83%), emphasizing the value of multivariate feature selection in capturing interactions among features. However, SelectKBest demonstrated higher stability (89.7%), indicating a trade-off between predictive performance and reproducibility that should inform practical implementation choices.

Despite these advances, several limitations warrant consideration. The CBIS-DDSM dataset, while widely used, is based on a specific demographic and imaging protocol, which may limit its generalizability. Its retrospective nature and curation process may introduce selection bias. Furthermore, evaluation on a single dataset restricts assessment of cross-domain robustness, and standard accuracy metrics may not fully reflect clinical utility, as false negatives carry greater consequences than false positives.

To address these challenges, future work should prioritize multi-institutional validation using federated learning frameworks. Such an approach would enable training and evaluation across diverse populations, imaging devices, and acquisition protocols, enhancing model robustness and equity. Additionally, integrating active learning strategies [33] could optimize expert annotation efforts by targeting high-uncertainty or outlier cases, thereby accelerating model refinement while minimizing labeling costs.

In conclusion, our findings highlight the importance of architectural depth, rigorous statistical validation, and multimodal feature design in developing reliable AI tools for breast cancer detection. While high accuracy is achievable, true clinical translation will require attention to generalizability, interpretability, and alignment with real-world screening workflows.

## 6. Conclusions

In this study, we proposed an integrated methodology for the early detection of breast cancer by combining data augmentation, multimodal feature selection, and the evaluation of deep learning models. Our results demonstrate the effectiveness of ResNet152, which achieved an accuracy of 97%. The robustness of our methodology was further validated by consistent performance improvements across various transfer learning models, including ResNet101, ResNet50, and VGG19, all of which achieved high accuracies. This highlights the versatility and strength of our approach to enhancing breast cancer detection across different deep learning architectures. Our methodology effectively combines radiomics with deep learning, providing a solid foundation for future research in medical image analysis and demonstrating the potential for improving early detection systems. While the results of this study are promising, there are several avenues for further research and improvement. A potential avenue for future research includes the use of Vision Transformers (ViTs), which have shown considerable success in computer vision tasks by employing self-attention mechanisms to identify long-range dependencies in images. Unlike traditional CNN-based models, ViTs treat image patches as sequences and can learn spatial hierarchies more effectively. By incorporating ViTs into our framework, we can explore their ability to enhance feature extraction, particularly in complex medical images like those used in breast cancer detection.

## Figures and Tables

**Table 1:** Description of the dataset distribution, including the count of original and augmented images for each class (benign and malignant).

Labels	Original Images	Augmented Images
Benign	1930	8498
Malignant	1354	8498
Total	3284	16,996

**Table 2:** Summary of feature selection methods and the number of selected features.

Method	Num_Features
RFE (Random Forest)	10, 20, 50, 100
RFE (Logistic Regression)	10, 20, 50, 100
RFECV (Random Forest)	74
RFECV (Logistic Regression)	647
SelectKBest (ANOVA)	10, 20, 50, 100
LASSO (LassoCV)	90
LASSO (LassoCV)	157
XGBoost GPU	50, 100, 200
LightGBM GPU	50, 100, 200
CatBoost GPU	50, 100, 200
Mutual Information	50, 100, 200

**Table 3:** Architectural and training configuration of pre-trained deep learning models used in the experimental evaluation. The table summarizes key specifications, including input dimensions, kernel sizes, architectural details, fine-tuned layers, optimizer, learning rate (LR), batch size, and regularization strategy (L2 penalty with λ=0.01), common across all models. All models were fine-tuned using the Adam optimizer with a learning rate of $10^{-5}$ and a batch size of 32.

Model	Input Size	Base Kernel Sizes	Models Architecture	Fine-Tuned Layers	Optimizer	LR	Batch Size	Regularization
VGG16	224×224×3	3×3 conv, 2×2 pool	13 conv + 3 FC (standard VGG)	Top 30%	Adam	$1\times 10^{-5}$	32	L2 (λ=0.01)
VGG19	224×224×3	3×3 conv, 2×2 pool	16 conv + 3 FC (standard VGG)	Top 30%	Adam	$1\times 10^{-5}$	32	L2 (λ=0.01)
ResNet50	224×224×3	7×7, 1×1, 3×3	50 layers; bottleneck blocks	Top 30%	Adam	$1\times 10^{-5}$	32	L2 (λ=0.01)
ResNet101	224×224×3	7×7, 1×1, 3×3	101 layers; bottleneck blocks	Top 30%	Adam	$1\times 10^{-5}$	32	L2 (λ=0.01)
ResNet152	224×224×3	7×7, 1×1, 3×3	152 layers; bottleneck blocks	Top 30%	Adam	$1\times 10^{-5}$	32	L2 (λ=0.01)
ResNet50V2	224×224×3	7×7, 1×1, 3×3	Improved pre-activation	Top 30%	Adam	$1\times 10^{-5}$	32	L2 (λ=0.01)
ResNet101V2	224×224×3	7×7, 1×1, 3×3	Improved pre-activation	Top 30%	Adam	$1\times 10^{-5}$	32	L2 (λ=0.01)
ResNet152V2	224×224×3	7×7, 1×1, 3×3	Improved pre-activation	Top 30%	Adam	$1\times 10^{-5}$	32	L2 (λ=0.01)
DenseNet121	224×224×3	7×7, 1×1, 3×3	121 layers; dense connections	Top 30%	Adam	$1\times 10^{-5}$	32	L2 (λ=0.01)
DenseNet169	224×224×3	7×7, 1×1, 3×3	169 layers; dense connections	Top 30%	Adam	$1\times 10^{-5}$	32	L2 (λ=0.01)
DenseNet201	224×224×3	7×7, 1×1, 3×3	201 layers; dense connections	Top 30%	Adam	$1\times 10^{-5}$	32	L2 (λ=0.01)
MobileNet	224×224×3	3×3 depthwise, 1×1 pw	Lightweight separable conv	Top 30%	Adam	$1\times 10^{-5}$	32	L2 (λ=0.01)
InceptionV3	299×299×3	1×1, 3×3, 5×5	Inception modules, factorized	Top 30%	Adam	$1\times 10^{-5}$	32	L2 (λ=0.01)

**Table 4:** Distribution of radiomics features by category.

Category	Number of Features
GLCM	264
GLSZM	176
GLRLM	176
First-order	198
GLDM	154
NGTDM	55
Shape	9
Diagnostics	5
Other	3

**Table 5:** Comprehensive comparison of radiomics feature selection methods [Ac = Accuracy, Prec = Precision, Reca = Recall, F1s = F1 Score, St = Stability].

Method	Features	Ac (%)	Prec (%)	Reca (%)	F1s (%)	St
RFE (RF)	100	83.70	86.20	80.00	83.00	0.485
RFECV (RF)	74	83.10	85.40	79.50	82.30	0.353
RFE (RF)	20	82.90	84.90	79.70	82.20	0.331
RFE (Random Forest)	50	82.80	84.70	79.70	82.10	0.403
RFE (Random Forest)	10	82.70	85.20	78.70	81.80	0.346
RFE (LR)	50	82.50	84.60	79.10	81.70	0.239
CatBoost GPU	50	82.40	85.50	78.80	82.10	0.313
SelectKBest (ANOVA)	100	82.40	85.60	77.50	81.40	0.766
SelectKBest (ANOVA)	20	82.20	83.50	79.80	81.60	0.897
LightGBM GPU	100	82.20	84.60	79.40	81.90	0.348

**Table 6:** Analysis of the performance characteristics of transfer learning models [Ac = Accuracy, Prec = Precision, Reca = Recall, Spec = Specificity, F1s = F1 Score, AUC = Area Under Curve, Ep = Epochs].

Model	Ac (%)	Prec (%)	Reca (%)	Spec (%)	F1s (%)	AUC (%)	Ep
**DenseNet169**	**95.0**	**94.0**	**94.0**	**96.0**	**95.0**	**98.80**	50
DenseNet201	94.0	93.0	94.0	96.0	94.0	98.90	50
DenseNet121	93.0	93.0	94.0	95.0	94.0	98.60	50
**ResNet152**	**97.0**	**97.0**	**98.0**	**96.0**	**97.0**	**99.30**	40
ResNet101	96.0	96.0	96.0	96.0	96.0	99.10	48
ResNet50	94.0	95.0	94.0	96.0	94.0	98.80	50
ResNet50V2	93.0	93.0	93.0	93.0	93.0	98.50	50
ResNet101V2	96.0	96.0	96.0	94.0	96.0	99.20	45
ResNet152V2	94.0	94.0	94.0	93.0	94.0	98.70	50
InceptionV3	89.0	89.0	88.0	91.0	90.0	97.50	50
MobileNet	88.0	87.0	86.0	89.0	88.0	97.00	50
**VGG19**	**96.0**	**96.0**	**96.0**	**96.0**	**96.0**	**99.00**	50
VGG16	94.0	94.0	94.0	94.0	94.0	98.50	29

**Table 7:** Model comparison results with 95% bootstrap confidence intervals and mean ranks [Ac = Accuracy, Prec = Precision, Reca = Recall, F1s = F1 score.]

Model	Ac (%)	Prec (%)	Reca (%)	F1s (%)	Mean Rank
VGG16	96 [94, 97]	96 [94, 97]	96 [94, 97]	96 [94, 97]	3.5
ResNet152	96 [94, 97]	96 [95, 97]	96 [95, 97]	96 [94, 97]	3.5
ResNet101	96 [94, 97]	96 [94, 97]	96 [94, 97]	96 [94, 97]	3.5
VGG19	95 [94, 97]	95 [94, 97]	95 [94, 97]	95 [94, 97]	6.0
ResNet50	95 [94, 97]	95 [94, 97]	95 [94, 97]	95 [94, 97]	6.0
DenseNet169	95 [93, 96]	95 [93, 96]	95 [93, 96]	95 [93, 96]	6.5
DenseNet201	95 [94, 97]	95 [94, 97]	95 [94, 97]	95 [94, 97]	6.0
ResNet101V2	95 [93, 96]	95 [93, 96]	95 [93, 96]	95 [93, 96]	6.5
ResNet50V2	94 [92, 95]	94 [92, 95]	94 [92, 95]	94 [92, 95]	8.5
DenseNet121	94 [92, 95]	94 [92, 95]	94 [92, 95]	94 [92, 95]	8.5
ResNet152V2	93 [92, 95]	93 [92, 95]	93 [92, 95]	93 [92, 95]	10.0
InceptionV3	91 [89, 93]	91 [89, 93]	91 [89, 93]	91 [89, 93]	11.5
MobileNet	87 [85, 89]	87 [84, 89]	87 [84, 89]	87 [85, 89]	13.0

**Table 8:** Per-class metrics for malignant class [Prec = Precision, Reca = Recall, F1s = F1 score].

Model	Prec (%)	Reca (%)	F1s (%)
VGG16	29.3	95.9	44.9
ResNet152	29.3	95.3	44.8

**Table 9:** Comparison of performance with recent studies using Deep Learning, and Transfer Learning. [Ac = Accuracy].

Author(s)	Methods	Ac (%)
Yu et al. [28]	ResNet34	72
	ResNet50	82
	VGG16	71
Gao et al. [31]	ResNet	82
Wei et al. [29]	ResNet50	72
	ResNet101	76
	VGG19	83
	Inception_v3	72
Sharmin et al. [30]	ResNet50V2	95
Yang et al. [32]	3DResNet	74
Alexandru et al. [19]	DenseNet121	99.6
Francesca et al. [21]	ResNet50	60
Wang et al. [22]	CNN	96.4
**Our Study**	ResNet50	94
	ResNet50V2	93
	ResNet101	96
	ResNet101V2	96
	ResNet152	97
	ResNet152V2	94
	VGG19	96
	VGG16	94
	Inception_v3	89

## Data Availability

The data can be accessed through the following link: https://www.cancerimagingarchive.net/, and the DOI is https://doi.org/10.7937/K9/TCIA.2016.7O02S9CY.
